# Three-Year Point Prevalence Survey of Antimicrobial Use in a Chinese University Hospital

**DOI:** 10.1155/2024/6698387

**Published:** 2024-02-08

**Authors:** Fa-Hong Jing, Qiang Wang, Tian-Jiao He, Na Xin, Yao-Wei Wang, Yang Han, Xin Wang, Zhuo Li

**Affiliations:** ^1^Department of Laboratory Medicine, The First Affiliated Hospital of Xi'an Medical University, Xi'an 710077, China; ^2^Department of Infection Management, The First Affiliated Hospital of Xi'an Medical University, Xi'an 710077, China

## Abstract

To evaluate the prevalence and quality of antimicrobial prescriptions using a Global Point Prevalence Survey (PPS) tool and help identify targets for improvement of antimicrobial prescribing and inform the development of antimicrobial stewardship activities. Antimicrobial prescriptions for inpatients staying at a hospital overnight were surveyed on one weekday in October 2018, November 2019, and November 2020. Data including basic patient information, antimicrobial drugs, quality evaluation of antimicrobial drug prescription, and the risk factors of nosocomial infection were collected from doctor network workstation. Patient information was anonymized and entered in the PPS Web application by physicians. A total of 720 patients (median age, 62 years) were surveyed. Of them, 246 (34.2%) were prescribed antimicrobials on the survey days. Hospital-wide antimicrobial use had a significantly decreasing trend (*P* < 0.001). The most commonly prescribed antimicrobial drugs were third-generation cephalosporins (40.5%), followed by quinolones (21.8%) and second-generation cephalosporin (12.5%). In our study, cefoperazone/sulbactam, ceftazidime, and levofloxacin were the most commonly used antimicrobials. The most common indication for antimicrobial use was pneumonia or lower respiratory tract infection (159/321, 49.5%). Antimicrobial for surgical prophylaxis represented 16.2% of the total antibiotic doses. Of those, 67.3% were administered for more than 24 h. The rate of adherence to antibiotic guidelines was 61.4%. The indications for antimicrobials were not documented in 54.5% of the prescriptions. Stop/review date was documented for 36.8% of prescriptions. The PPS tool is useful in identifying targets to enhance the quality of antimicrobial prescriptions to improve the adherence rate in hospitals. This survey can be used as a control to assess the rational application quality of antimicrobial after regular application of antimicrobial intervention.

## 1. Introduction

Antimicrobial resistance has been a global hazard problem for the past two decades. It has been attributed to the abuse of antimicrobials, particularly broad-spectrum antimicrobials, in both in-hospital and outpatient settings. Nearly up to 40% of hospitalized patients receive antimicrobial prescriptions, which are noncompliant with clinical guidelines. Additionally, the excessive use of antimicrobials for inappropriate indications or incomplete durations can also increase the burden of antimicrobial resistance. Point prevalence studies can determine the areas of misuse and provide guidance in developing national strategies for antibiotic use.

Antimicrobial-resistant pathogens and the lack of newly developed antimicrobials have become global concerns [1]. It requires coordinated action at the local, national, and global levels [2]. After the World Health Organization began to emphasize these problems in 2011, many countries started to seek solutions. In 2012, the China's Ministry of Health issued Decree No. 84 “Administrative Measures for Clinical Application of Antimicrobial,” which marked the legalization and institutionalization of the management of clinical application of antimicrobial in China. It is important for hospitals to monitor antimicrobial use, learn about hospital-associated infections, and detect microbial pathogens. However, most studies use random sampling, which leads to inaccurate and untrue results. One of the most commonly used standard PPS protocols was developed by the PPS protocols European Centre for Disease Prevention and Control (ECDC) [3]. Besides the ECDC-PPS, the Global Point Prevalence Survey of Antimicrobial Consumption and Resistance (Global-PPS) was organized by the University of Antwerp to monitor the ratios of antimicrobial prescribing and resistance in hospitalized inpatients on a worldwide level, with special attention to low- and middle-income countries. The Point Prevalence Survey (PPS) is a widely used cross-sectional investigation and research, which aims to describe hospital data, especially the description of healthcare-associated infections (HAIs)/pathogenic microorganisms and antimicrobials. PPS can provide a basis for refining the management of antimicrobial and drug resistance and formulating relevant intervention strategies. Regular PPS can be used to determine changes in antimicrobial use of hospitals in order to identify and address existing problems and evaluate the effectiveness of improvement measures. Large national and multinational surveys have been performed recently in most of the countries [4, 5]. Local, regional, and national surveys were also organized in the People's Republic of China [6, 7].

## 2. Materials and Methods

### 2.1. Study Design and Setting

One-day PPS were performed annually at the First Affiliated Hospital of Xi'an Medical University. Each PPS from 2018 to 2020 took place for each of the three survey periods (October 2018, November 2019, and November 2020, hereinafter referred to as first, second, and third period, respectively). Patients who were present at 8 a.m. on the day of the survey in certain departments were included in the study. A patient list was retrieved from electronic patient records. Data were collected by doctors and pharmacists who reviewed their medical records, while nurses collected data from medical devices.

### 2.2. Data Collection

Inpatients hospitalized at 8 a.m. on the day of the survey were included. In 2018, the investigation departments were the respiratory department, hematology department, neurology department, general surgery department, neurosurgery department, and orthopedics department; in 2019, the respiratory department, orthopedics department, and respiratory and critical medicine department; and in 2020, the endocrinology department, general department, neurology department, and oncology department. Patient-level information on antimicrobial prescription was collected, except for those of outpatient patients, patients discharged before 8 a.m., and those admitted with intervention after that study time. Further details on the Global-PPS protocol have been described elsewhere [8]. The medical data of patients who were prescribed antimicrobials were obtained from the hospital pharmacy in spreadsheet format, verified against the medical records, and extracted by physicians from the Global-PPS surveillance sheet. The data included the patients' characteristics and details of their prescribed antimicrobials, such as drug name, unit dose, frequency, and reasons for prescribing. Compliance with clinical guidelines was also measured by assessing the prescribing patterns against institutional antimicrobial prescribing guidelines. The results were reported for three points separately. The frequency of antimicrobial use was estimated as the ratio of the number of patients prescribed antimicrobials to the total number of inpatients in each surveyed ward. Antimicrobials were classified based on the World Health Organization (WHO) Anatomical Therapeutic Chemical Classification code system [9].

### 2.3. Data Analysis

The frequency of antimicrobial use was determined by calculating the percentages of patients taking at least one antimicrobials relative to the total number of admitted patients on the day of the PPS. The proportion of antimicrobials prescribed based on treatment indication and diagnosis was calculated. Agreement with quality indicators was denoted by a percentage of the total number of prescribed antimicrobials. For treatment based on biomarker data or microbiology laboratory test results, the denominator was the number of antimicrobials prescribed for therapeutic use [10]. Categorical variables were reported as percentages. For frequency of antimicrobial use and quality indicators, the trend over time was assessed using the chi-squared test. A *P* value of 0.005 was considered statistically significant. Data were extracted in Microsoft Excel and analyzed using SPSS 25.

## 3. Results

### 3.1. Demographics and Clinical Characteristics of Inpatients

A total of 720 patients (median age, 62 years; interquartile range [IQR], 26–88) were included in this study. The demographics and clinical characteristics of inpatients for the 3 years of PPS are described in Table 1. On the survey days, the average bed occupancy rate was 91%. Device insertion rates for central venous, peripheral venous, urinary catheters, and tracheal/tracheostomy tubes were 3.3%, 10.1%, 10.4%, and 4.0%, respectively. In the majority of cases in three years, the antimicrobials were administered empirically (92.3%, 87.3%, and 58.7%, respectively) and were rarely based on biomarker levels (16.8%, 22.8%, and 58.7%, respectively). However, the indicators related to targeted therapy and treatment based on biomarker data showed a significantly increasing trend (*P* < 0.001). Of the total antibiotic doses, 16.2% were antimicrobials for surgical prophylaxis, of which 67.3% were administrated for more than 24 h.

### 3.2. Frequency of Antimicrobial Use

Over the three survey periods, 720 patients were admitted due to different indications, and a total of 321 antimicrobials were prescribed to 246 inpatients. The overall hospital-wide antimicrobial use was 34.2%, 27.2% of patients received a combination of two antimicrobials, and 1.6% of patients used three or more antimicrobials (Table 2). The hospital-wide antimicrobial use was 35.5% in the first period, 55.6% in the second period, and 18.6% in the third period, demonstrating a significantly decreasing trend for hospital-wide antimicrobial use (*P* < 0.001).

### 3.3. Frequency of Main Antimicrobial Classes

The use of the main antimicrobial classes was also evaluated and described in Table 3. Third-generation cephalosporins (40.5%) were the most commonly prescribed antimicrobial groups, followed by quinolones (21.8%) and second-generation cephalosporin (12.5%).

### 3.4. Reasons for Antimicrobial Prescriptions

**Figure 1:**
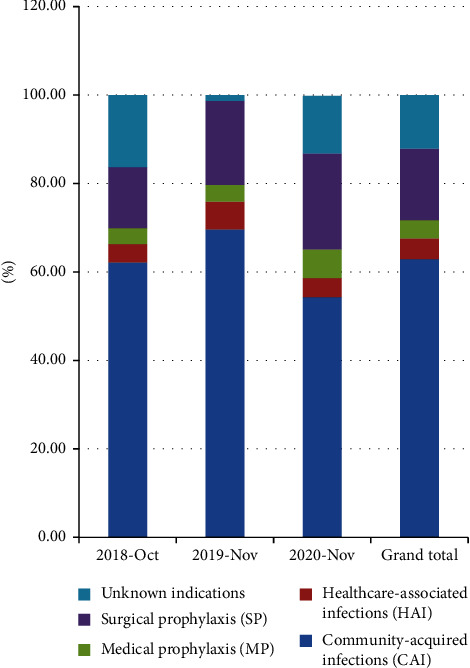
Indications for antimicrobial prescribing (percentage).

In the 321 antimicrobial prescriptions, 15 (4.7%) were used for HAIs, 202 (62.9%) for community-acquired infections, 52 (16.2%) for surgical antibacterial prophylaxis, and 13 (4.1%) for medical prophylaxis (Figure 1).

The most common indication for antimicrobial prescription was pneumonia or lower respiratory tract infection, with 159 prescriptions (49.5%), while the least prevalent indication was both ear, nose, and throat infection and intra-abdominal infection (Table 4).

### 3.5. Quality Indicators of Antimicrobial Prescriptions

**Figure 2:**
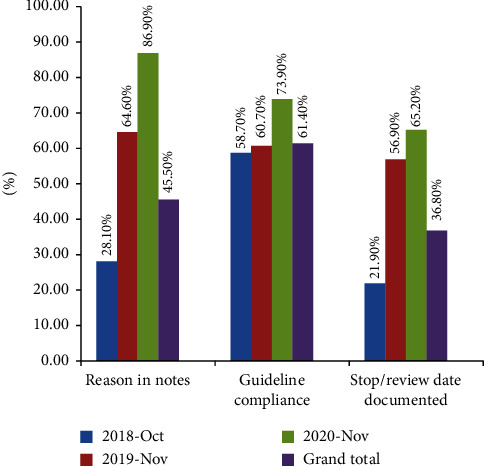
Quality indicators for antimicrobial prescriptions during three survey periods.

Based on the analysis results of the survey, 45.5% of antimicrobial prescriptions were justified per medical records, and 36.8% had a stop/review date documented; guidelines compliance for all antimicrobial prescriptions was 61.4%. The quality indicators for antimicrobials prescription are shown in Figure 2. As regards compliance with clinical guidelines, most of the antimicrobial prescriptions were compliant to guidelines. A significantly increasing trend was observed for the quality indicators of antimicrobial prescriptions.

## 4. Discussion

The overall antimicrobial use prevalence observed in this study was 34.2%, which was similar to the 2015 global data (34.4%), the regional data for East and South Asia (37.5%) [8, 10]. From 2018 to 2020, the frequency of antimicrobial use in our hospital was 35.5%, 55.6%, and 18.6%, respectively, demonstrating an obvious downward trend but with significant differences between them. This might be due to the different investigation departments selected in the three years. Due to a large number of critically ill patients in respiratory ICUs in 2019, the use of antimicrobials was significantly increased. The frequency of antimicrobial use in the present study was 55.6%, which was basically the same as Shanxi Province and that of 13 other hospitals in China [6, 11]. In recent years, our hospital adheres to the *Guiding Principles for Clinical Application of Antibiotics (2015 Edition)*, *the Management Measures for Clinical Application of Antibiotics* and other relevant documents, and formulates monthly reviews of antibacterial drug prescriptions and rational use of perioperative prophylactic drugs to promote the rational use of antibiotics. The 3-year data showed improvement in some clinical indicators, which may be related to the clinical feedback on relevant results after each PPS. This feedback may prompt doctors to take measures to improve the rational use of antimicrobial drugs (e.g., patient intubation rate and target treatment rate).

In this study, a significant proportion of antimicrobials was used to treat community-acquired infections (CAI) (62.9%), which was similar to the results of a study in India [12]. This means that CAI is an indicator of the highest prevalence of antimicrobial use [13]. Medication use for either CAI or hospital-acquired infection was associated with a lower rate of target therapy (7.7%, 12.7%, and 41.3%) and was dominated by empirical medication and surgical premedication, which was similar to results in other countries, such as Europe and the United States [14, 15]. This may be due to the low effective culture rate of microorganisms [16], attributed to low specimen delivery rate and noncompliance. On the other hand, it may be due to the severity of the patient's condition, which requires first empirical treatment to control the condition as well as appropriate adjustment after the return of bacterial culture and susceptibility results. Based on our findings, the target treatment rate had been on the rise in the past 3 years, which may be related to the hospital's continuous strengthening of training and emphasis on rational use of antimicrobial. In our study, cefoperazone/sulbactam, ceftazidime, and levofloxacin were the most commonly used antimicrobials, similar to those used in European countries [17]. This is mainly because approximately half of the diagnoses of infection in this study were pneumonia or lower respiratory tract infection, which is similar to many countries worldwide. However, ceftriaxone is rarely used. The differences in patterns of antimicrobial use between the investigated hospitals may be related to the degree of regional bacterial resistance, the level of guideline implementation, the degree of empirical treatment, the diversity of prescribing practices, and the variety of antimicrobial species introduced.

Indications for antimicrobial prescription were mainly classified as HAI (4.9%), CAI (62.9%), MP (4.1%), SP (16.2%), and OTH (12.1%). Annual trends in the prevalence of HAIs showed no significant changes, which are consistent with 8 years of PPSs in Chinese hospitals (5.03% in 2010-2011 and 5.04% in 2016-2017) [18]. A European PPS study found that country-weighted HAI prevalence before validation correction in acute-care hospitals was 5.7% in 2011-2012 and 5.5% in 2016-2017 [19]. However, the factors for the relatively low HAI prevalence in China are not clear. In certain high-risk groups of bacterial infections, medical prophylaxis can be used, for example, severe neutrophil deficiency (ANC in 2011^9^/L) high-risk patients or patients undergoing solid organ transplantation and hematopoietic stem cell transplantation with a duration exceeding 7 days. International guidelines on surgical prophylaxis recommend that a single dose of narrow-spectrum antimicrobial be administered within the 24-h preoperative period [20, 21]. Of the total antibiotic doses, 16.2% of antimicrobials were for surgical prophylaxis, of which 67.3% were administrated for more than 24 h and often involved the use of broad-spectrum antimicrobials, such as cefuroxime and ceftazidime [22]. This finding provides an opportunity to optimize the use of systemic AMDs and to reduce the duration of perioperative antimicrobial prophylaxis based on the recommended single doses because the prolongation for at least 1 day does not prevent infectious complications but increases the risk of antimicrobial resistance and adverse events including acute kidney injury and *Clostridioides difficile* infection [23]. Therefore, the management of prophylactic drugs in surgery should be strengthened.

Quality indicators for antimicrobial prescribing included reasons for prescriptions in notes, guideline compliance, and stop/review documentation. In general, the medical records were detailed with regard to medical treatment steps, selection of antimicrobial agents, and administration start criteria, but there were few records with a stop criterion or review, and the status of termination was unclear. Documentation of a stop/review date rate was low, at only 36.8% of all prescriptions. In the 2015 global analysis, 38.3% of the antimicrobial prescriptions globally and 19.8% of prescriptions in East and South Asia had a recorded date for stopping or reviewing the antimicrobial regimen [8]. Suboptimal documentation has been reported in a number of hospitals, as compared with reported on quality indicators in other countries [24]. In our hospital, the quality evaluation index of antimicrobial drugs steadily improves, owing to the quality control department's attention and in-depth management.

This study has some limitations. Firstly, its design is purely observational, not controlled, and does not use interrupted time series analyses; such analysis method is considered more robust in evaluating the impact of AMS interventions and long-term trends in antimicrobial prescription. A decrease in in-hospital antimicrobial use has been reported after 2 years of intervention and regulation, particularly for two quality indicators on the documentation of antibiotic prescriptions (i.e., stop/review date and reason for prescription), both of which had exceeded their predefined target of 85% [25]. Secondly, our data were difficult to compare because of the small population included in the survey and the different departments surveyed each year.

## 5. Conclusions

In conclusion, the study presents data on patient background, antimicrobial prescription details, the quality evaluation of antimicrobial drug prescriptions, and the analysis of risk factors of nosocomial infections. The PPS tool is useful in identifying targets to enhance the quality of antimicrobial prescriptions to improve the adherence rate in hospitals. In a further study, we intend to conduct repeated PPS measurements to track the changes in antimicrobial prescription in order to generate consecutive results on sustained behavioral changes in the prescription practice in our hospital. Based on the study, we should focus on improving the standardization of surgical perioperative prescriptions, including labeling of antibiotic usage time, dosage, and withdrawal time.

## Figures and Tables

**Table 1:** Demographics and clinical characteristics of inpatients for the 3 years of PPSs.

Year	2018-Oct	2019-Nov	2020-Nov	Grand total	*P* value
Sex, male, *N* (%)	225 (55.0)	86 (73.5)	115 (59.3)	426 (59.2)	
Number of patients, *N*	409	117	194	720	
Bed occupancy rate in total, (%)	95.6	88.6	88.9	91	
Age, median, (IQR)	62 (26–85)	61 (28–78)	62 (31–88)	62 (26–88)	

*Patients with devices in place, N (%)*					
CVC/CV port/PICC	15 (3.7)	7 (5.9)	2 (1.0)	24 (3.3)	0.053
PVC	38 (9.3)	26 (22.2)	9 (4.6)	73 (10.1)	<0.001
Urinary catheter	45 (11.0)	14 (12.0)	16 (8.2)	75 (10.4)	0.489
Tracheal-tracheostomy tube	21 (5.1)	4 (3.4)	4 (2.1)	29 (4.0)	0.188
Number of prescriptions, *N*	196	79	46	321	
Targeted therapy, *N* (%)	15 (7.7)	10 (12.7)	19 (41.3)	44 (13.7)	<0.001
Treatment based on biomarker data, *N* (%)	33 (16.8)	18 (22.8)	27 (58.7)	78 (24.3)	<0.001

*Surgical antibiotic prophylaxis*					
Antimicrobials for surgical prophylaxis, *N* (%)	27 (13.8)	15 (19.0)	10 (21.7)	52 (16.2)	0.310
Antimicrobials for surgical prophylaxis of ≤1 day, *N* (%)	8 (29.6)	5 (33.3)	4 (40.0)	17 (32.7)	0.835
Surgical prophylaxis (single dose), *N* (%)	23 (85.2)	6 (40.0)	5 (50.0)	34 (65.4)	0.007
Guideline compliance, *N* (%)	25 (92.6)	12 (80.0)	8 (80.0)	45 (86.5)	0.413

CV, central venous; CVC, central venous catheter; PICC, peripherally inserted central catheter; PVC, peripheral venous catheter; IQR, interquartile range.

**Table 2:** Use of antimicrobial in PPS days.

Time	Number of patients	Antibiotic use rate	Single	Duplex	Triplicate	Number of prescriptions
2018-Oct	409	145 (35.5%)	97 (66.9%)	45 (31.0%)	3 (2.1%)	196
2019-Nov	117	65 (55.6%)	52 (80.0%)	12 (18.5%)	1 (1.5%)	79
2020-Nov	194	36 (18.6%)	26 (72.2%)	10 (27.8%)	0 (0.0%)	46
Grand total	720	246 (34.2%)	175 (71.1%)	67 (27.2%)	4 (1.6%)	321
*P* value		<0.001	0.151	0.166	0.678	

**Table 3:** Prevalence of main antimicrobial classes.

Antimicrobial	2018-Oct *N* (%)	2019-Nov *N* (%)	2020-Nov *N* (%)	*P* value
Third-generation cephalosporin (J01DD)	67 (34.2)	37 (46.8)	26 (56.5)	0.009
Quinolones (J01M)	49 (25.0)	13 (16.5)	8 (17.4)	0.220
Second-generation cephalosporin (J01DC)	31 (15.8)	5 (6.3)	4 (8.7)	0.069
Other antibacterials (J01X)	18 (9.2)	2 (2.5)	1 (2.2)	0.056
Antimycobacterials for systemic use (J02A)	9 (4.6)	0 (0.0)	1 (2.2)	0.129
Macrolides/lincosamides/streptomycin	6 (3.1)	1 (1.3)	0 (0.0)	0.359
Nitroimidazoles (P01A)	6 (3.1)	1 (1.3)	3 (6.5)	0.264
Carbapenems (J01DH)	4 (2.0)	6 (7.6)	1 (2.2)	0.064
First-generation cephalosporins (J01DB)	3 (1.5)	10 (12.7)	2 (4.3)	<0.001
Aminoglycoside antibiotics (J01G)	2 (1.0)	3 (3.8)	0 (0.0)	0.159
Tetracyclines (J01A)	1 (0.5)	1 (1.3)	0 (0.0)	0.652

**Table 4:** Diagnoses treated with therapeutic antimicrobials (%).

Indication	2018-Oct *N* (%)	2019-Nov *N* (%)	2020-Nov *N* (%)
Pneumonia or lower respiratory tract infection	89 (45.4)	48 (60.8)	22 (47.8)
Skin and soft tissue infection	18 (9.2)	2 (2.5)	2 (4.3)
Urinary tract infection	10 (5.1)	2 (2.5)	1 (2.2)
Ear, nose, and throat infection	3 (1.5)	1 (1.3)	0 (0.0)
Bronchitis	10 (5.1)	10 (12.7)	1 (2.2)
Intra-abdominal infection	1 (0.5)	0 (0.0)	0 (0.0)
Bone/joint infection	16 (8.2)	13 (16.5)	6 (13.0)
Gastrointestinal infection	6 (3.1)	1 (1.3)	1 (2.2)
Obstetric/gynecological infection	5 (2.6)	0 (0.0)	5 (10.9)
Infection of the central nervous system	6 (3.1)	1 (1.3)	2 (4.3)
Unknown indications	32 (16.3)	1 (1.3)	6 (13.0)

## Data Availability

The study data used to support the findings of this study are included within the article.
